# On Fever

**Published:** 1829-01-01

**Authors:** 


### CORK-STREET FEVER HOSPITAL.

FEVER.—We have received an official medical report (printed) from the above institution, apparently drawn up, for the Committee of Management, by the junior physician, Dr. O'Reardon. We shall extract some particulars from this document, for the benefit of our readers.

Dr. O'Reardon treated 803 cases of fever during the year 1827. He calculated, that about six-sevenths of the continued fevers were "complicated with various kinds of visceral affection," namely, inflammations, more or less acute, of the head, lungs, fauces, stomach, or intestines—or with rheumatism. The cephalic, pulmonary, and gastric complications were the most numerous and common. Our reporter had under his care two patients—each severely afflicted with a complication of dysentery and melæna. The repeated losses of blackish coagulated blood, of very offensive odour, completely blanched these two patients, and caused considerable languor. The following remedy was prescribed.

℞. Ol. ricini...... ʒvj.

Ol. terebinth... ℥ss.

Mucilag. acaciæ ʒiss.

Tinct. opii .... ʒss.

Syrupi........ ℥ss.

Misce fiat mistura, capiat quartam partem tertiis horis, ad quartem vicem.

This remedy was daily repeated for three or four days, by which the melæna was cured, and the dysentery yielded afterwards to a few doses of the oleum ricini.

"In the fully formed common Fever of these countries we observe an elevated temperature of body, acceleration of pulse, some degree of head-ach; foulness or rawness of tongue, not unfrequently enlargement of this organ, sometimes thickness of it, at other times a parched, glossy, or a dry rough chopped state of it, resembling a thinly incrusted blood-oozing sore; face either flushed or of a soiled dusky aspect, and of a semi-bloated appearance in the early stage of Fever; respiration somewhat accelerated and oppressed; nausea, thirst, costiveness; epigastrium full, and over sensible to the tact; liability to general abdominal soreness and inflammation: aching of various parts of the body, namely, of the spine, joints, thighs, and arms; a sense of general lassitude and heaviness: a state either of restlessness or somnolency. Such a condition assuredly belongs to a general internal subinflammatory action of the system, an action like all inflammatory movements mainly depending on, and kept up by an alteration and derangement of the circulation. When Fever is high the general subinflammatory movement in question often assumes a more decidedly inflammatory turn; and if it be not complicated from the beginning, in the progress of the great majority of cases it becomes extended to one two or more of the organs of the head, thorax, and abdomen, or to the muscular fibres, their membranes, and tendinous aponeuroses, so as to cause rheumatism.

"Combined with the general subinflammatory, or inflammatory state in question, there usually is a nervous condition, which modifies the Fever in some degree. We likewise often observe symptoms of vitiated bile and disordered functions of the liver and stomach, probably arising from the increased temperature of the body, and the altered state of the circulation, and re-acting on the Fever so as to produce corresponding modifications thereof. Many of our patients are seized at the same time with general pyrexia and visceral inflammation. In some other instances the local disorder is the primary complaint, attended with what is commonly termed Symptomatic Fever. The former course of attack belongs to the genus which is the leading subject of this paper, viz. to general continued Fever, embracing its complications. The latter might in like manner be classed as a species of the same genus."

The reporter conceives that, in a nosological table, as well as in the mind of the practitioner, "the febres and the phlegmasiæ should belong to one order."

"It is often difficult, and not always necessary, to ascertain exactly whether the priority of invasion belongs to the general Fever, or to the visceral affection. Some Fevers commence with symptoms of sanguineous plethora or congestion in the head; some with a deranged condition of the stomach, and especially of its mucous membrane and glands, and of the continuation of said membrane to the fauces and tongue. The stomach seldom fails to sympathize strongly with the head, when the latter suffers from severe fixed Cephalœa, caused by vascular turgescence or inflammation.

"Some Fevers begin with, or are from the commencement attended by, either sub-inflammation or complete inflammation of a part or the entire of the thoracic viscera; others with inflammation of the liver or bowels, or of some other part in the abdomen: others again with rheumatism. Whether the Complicatæ be simultaneous with or secondary or anterior to the general Fever, the ratio medendi is nearly or entirely the same. It is, however, quite essential in practice to ascertain as well as possible the organ or organs principally diseased; as by the early employment of the most appropriate means for the cure thereof, we shall be the better enabled to preserve our patient's lives, and abridge the duration of their Fevers."

The reporter considers the various divisions of fever, comprehended under the terms synocha, synochus, typhus mitior, typhus gravior, and twenty other designations, as "mere varieties of one genus —that is, of the above described general sub-inflammatory condition, constituting fever—varieties produced by the diet, occupation, locality, temperament, habit of body, state of mind, or nervous condition of the patient—or by an alteration in his biliary or other secretions—by the period of the fever or its treatment—by the season of the year, the climate, or constitution of the atmosphere."

The foregoing theory, or rather pathology, of fever removes, so thinks the reporter, that obscurity and confusion which long prevailed—and, in some places, still exists in the management of fever.

"It admits and requires the utmost attention in distinguishing the varieties and successive changes of Fever, and in discriminating the character of each disordered viscus. It indicates a rational, clear, effective, and tolerably uniform line of practice in the treatment of Fevers, and their manifold complications—a practice which the best modern physicians are for these many years back gradually adopting, as if it were by common consent."

This practice consists mainly in the antiphlogistic plan of treatment, modified according to circumstances. "It admits of the mildest practice in the mild cases, as well as of the most active treatment in severe cases." In whatever form the disease begins, in the advanced stages it becomes occasionally marked by "adynamic spasmodic nervous symptoms, with a black or blackish tongue, and a very peculiar alteration of countenance." Under these circumstances, indications arise which require all the mental resources of the practitioner.

"The Methodus Antiphlogistica, is to be employed not only in a very mitigated manner, but it must be combined with, and often superseded by antispasmodic, antinervous, diffusively stimulating and tonic remedies. As in the class of tonic medicines, cinchona holds a prominent station, I think it right to observe that this article is scarcely ever useful, and is often injurious in continued Fever during the existence of a hot skin, a quick pulse, and a loaded tongue; cases of gangrene excepted, where indeed the temperature is in general any thing but high, and where cinchona aided by camphorated mixture, good wine, and a suitable position of the body, is an extremely valuable curative agent. I have already noticed the great efficacy of this bark in Erysipelas, after the previous subtraction of some blood from the arm."

The opinion of every man, grounded on such a field of experience as a Fever Hospital, is entitled to respect, especially when it is accompanied by the salutary modifications and precautions contained in the foregoing extracts. On this account we shall not append any comments on the report, but allow the arguments of the Reporter to acquire all the weight to which they may be found entitled.

---

